# *Bursaphelenchus xylophilus* and *B. mucronatus* secretomes: a comparative proteomic analysis

**DOI:** 10.1038/srep39007

**Published:** 2016-12-12

**Authors:** Joana M. S. Cardoso, Sandra I. Anjo, Luís Fonseca, Conceição Egas, Bruno Manadas, Isabel Abrantes

**Affiliations:** 1 CFE - Centre for Functional Ecology, Department of Life Sciences, University of Coimbra, Calçada Martim de Freitas, 3000-456 Coimbra, Portugal; 2 Faculty of Sciences and Technology, University of Coimbra, 3030-790 Coimbra, Portugal; 3 CNC - Center for Neuroscience and Cell Biology, University of Coimbra, 3004-517 Coimbra, Portugal

## Abstract

The pinewood nematode, *Bursaphelenchus xylophilus*, recognized as a worldwide major forest pest, is a migratory endoparasitic nematode with capacity to feed on pine tissues and also on fungi colonizing the trees. *Bursaphelenchus mucronatus*, the closest related species, differs from *B. xylophilus* on its pathogenicity, making this nematode a good candidate for comparative analyses. Secretome profiles of *B. xylophilus* and *B. mucronatus* were obtained and proteomic differences were evaluated by quantitative SWATH-MS. From the 681 proteins initially identified, 422 were quantified and compared between *B. xylophilus* and *B. mucronatus* secretomes and from these, 243 proteins were found differentially regulated: 158 and 85 proteins were increased in *B. xylophilus* and *B. mucronatus* secretomes, respectively. While increased proteins in *B. xylophilus* secretome revealed a strong enrichment in proteins with peptidase activity, the increased proteins in *B. mucronatus* secretome were mainly related to oxidative stress responses. The changes in peptidases were evaluated at the transcription level by RT-qPCR, revealing a correlation between the mRNA levels of four cysteine peptidases with secretion levels. The analysis presented expands our knowledge about molecular basis of *B. xylophilus* and *B. mucronatu*s hosts interaction and supports the hypothesis of a key role of secreted peptidases in *B. xylophilus* pathogenicity.

The pinewood nematode (PWN), *Bursaphelenchus xylophilus*, an European Plant Protection Organization A2 pest^1^, is an invasive species responsible for the development of the pine wilt disease (PWD) and is recognized worldwide as a major forest pest. Although it is considered a native species of North America, American conifers are tolerant or resistant to PWN and, consequently, this nematode has caused little damage in this region^2^,^3^. At the beginning of the 20^th^ century, PWN was introduced in Japan and became responsible for massive mortality of native pine trees (*Pinus densiflora, P. thunbergii* and *P. luchuensis*) and then spread to China, Korea and Taiwan^3^. In Europe, it was first detected in continental Portugal in maritime pine, *P. pinaster*^4^ and more recently in *P. nigra*^5^. It has also been found in Madeira Island^6^ and Spain^7^,^8^ associated with *P. pinaster*, representing an increasing threat to European conifer forests. Prevention of *B. xylophilus* spread is particularly difficult as this nematode is vectored by bark beetles mainly belonging to the genus *Monochamus*^9^.

The international ecological and economic impact caused by the PWN highlight the need for further investigations on PWN pathogenic mechanisms which are still not clear. *Bursaphelenchus xylophilus* is a migratory endoparasitic nematode with the capacity to feed on pine tissues and also on fungi colonizing the tree. Nematodes feed on parenchyma cells and migrate in xylem tissues, spreading throughout the tree. This causes cell destruction, leading to wilting symptoms that results in the tree death within a few months. In the last few years, progress has been made in understanding the nature of proteins used by the PWN to successfully invade and feed on trees. Much of this progress has been supported by large-scale expressed sequence tag (EST) transcriptomic projects undertaken for *B. xylophilus*^10^,^11^,^12^ and also using comparative analyses for *B. xylophilus* and the closest related species, *B. mucronatus*^13^,^14^. *Bursaphelenchus mucronatus* is distributed throughout the Northern Hemisphere and is a prevalent species in Central and North Europe^15^. Although very similar in morphological and ecological characteristics, *B. xylophilus* and *B. mucronatus* are different in pathogenicity. *Bursaphelenchus mucronatus* was described to have weak pathogenicity and able to kill only trees exposed to severe stress^16^. The higher pathogenicity of *B. xylophilus* has been associated to its higher competitive potential and invasiveness. The higher reproductive ability results in a rapid population growth rate which allows it to colonise new habitats more easily than *B. mucronatus*^17^. Also migration and pine cell destruction abilities are important factors affecting these two species different pathogenicity and recent studies showed that the number and area of dead epithelial cells in pine cuttings inoculated with *B. mucronatus* were smaller than in those inoculated with *B. xylophilus*, suggesting that the attacking ability of *B. mucronatus* is weaker than that of *B. xylophilus*^18^. Thus, these two species are usually studied together for comparative analyses. A draft genome sequences of *B. xylophilus* were also reported in 2011^19^. The availability of all these comprehensive data sets accelerated the postgenomic studies on PWD and a large-scale proteomic study has been conducted to better understand the pathogenicity of *B. xylophilus*^20^. This study presented a complete profile of the *B. xylophilus* secretome, however, no secretome data for *B. mucronatus* was yet available.

In the present study, SWATH-MS was used to determine changes in protein amounts between *B. xylophilus* and *B. mucronatu*s secretions, bringing new insights into the molecular basis of these nematodes interaction with their hosts and PWN pathogenicity.

## Results

### Transcriptomic profiles

The entire reads set obtained for *B. mucronatus* transcriptome and used for the final assembly was submitted to the EMBL-EBI European Nucleotide Archive (ENA), under the study accession number PRJEB14884. A total of 465,256 raw pyrosequencing reads of a mean length of 337 bp were obtained for *B. mucronatus*. These were assembled in 8,822 contigs with a mean length of 766 bp. A total of 9,231 translated amino acid sequences were deduced from contigs sequences. Annotation of the contigs resulted in 5,547 peptides associated to InterPro protein families or functional domains and 4,067 peptides assigned to gene ontology (GO) terms.

**Figure 1:**
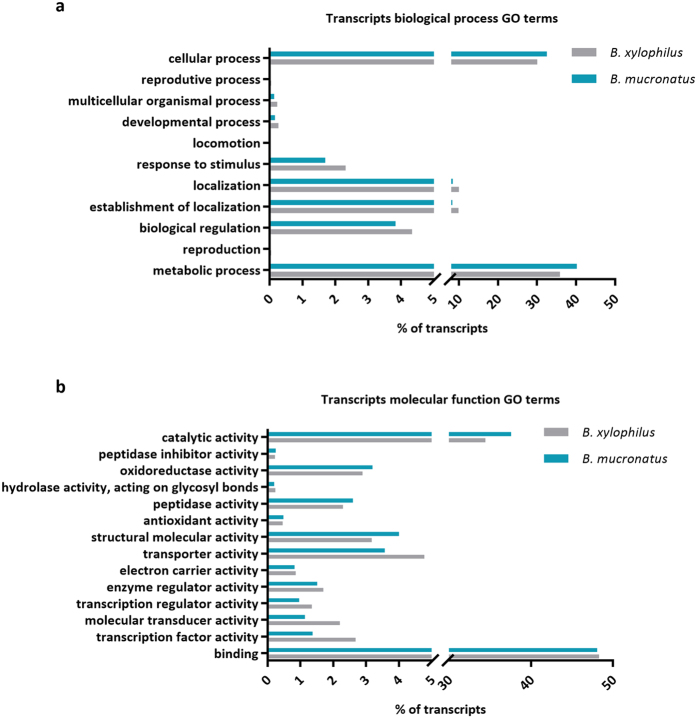
Distribution of *Bursaphelenchus xylophilus* and *B. mucronatus* transcripts according to gene ontology (GO) terms. Biological process (**a**) and molecular function (**b**).

Gene ontology analysis of *B. mucronatus* and *B. xylophilus* (Bioproject PRJNA192936) transcriptomes revealed that both nematodes have a similar composition, with a higher percentage of transcripts associated with cellular and metabolic processes in biological process GO category (Fig. 1a) and binding and catalytic activity in molecular function GO category (Fig. 1b). Analysis of higher levels of molecular function GO terms revealed that both nematodes also have similar composition on transcripts putatively related to pathogenicity such as peptidase activity and hydrolase activity, acting on glycosyl bonds (Fig. 1b).

### Proteomic profiles

**Figure 2:**
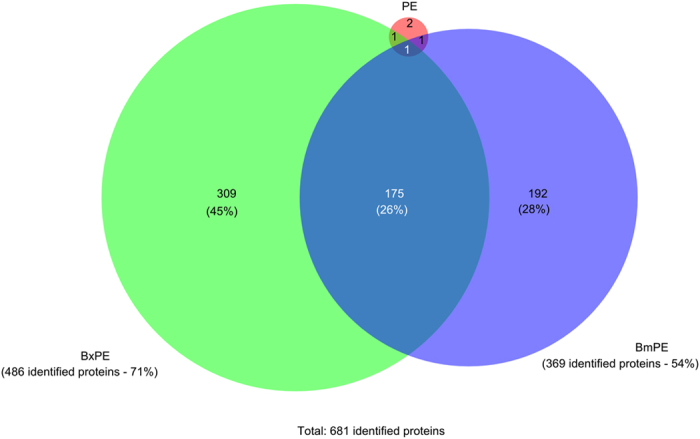
Venn diagram showing the distribution of identified proteins after information-dependent acquisition (IDA) experiments using the transcriptomic derived database. *Bursaphelenchus xylophilus* secretome (BxPE), *B. mucronatus* secretome (BmPE) and pine extract (PE). Protein identifications were obtained by combining the results of three pooled samples of each condition.

*General description and global results.* From information-dependent acquisition (IDA) experiments, secretome profiles of *B. xylophilus* (BxPE) and *B. mucronatus* (BmPE) were obtained, using either an annotated *B. xylophilus* protein database derived from genome data (BioProject PRJEA64437)^19^ or using a combined database derived from the transcriptomic data of *B. xylophilus* and *B. mucronatus*. A total of 520 proteins were identified in the three experimental conditions BxPE, BmPE and PE (negative control) using the genomic database (Supplementary Table S1 and Fig. S1), while a total of 681 proteins were identified in the three conditions using the transcriptomic derived database (Supplementary Table S2), with an overlap of 50% between the two databases (Supplementary Table S3 and Fig. S2). In BxPE condition a higher number of proteins were identified compared to the BmPE condition and, as expected, fewer proteins were identified in PE (Fig. 2).

**Figure 3:**
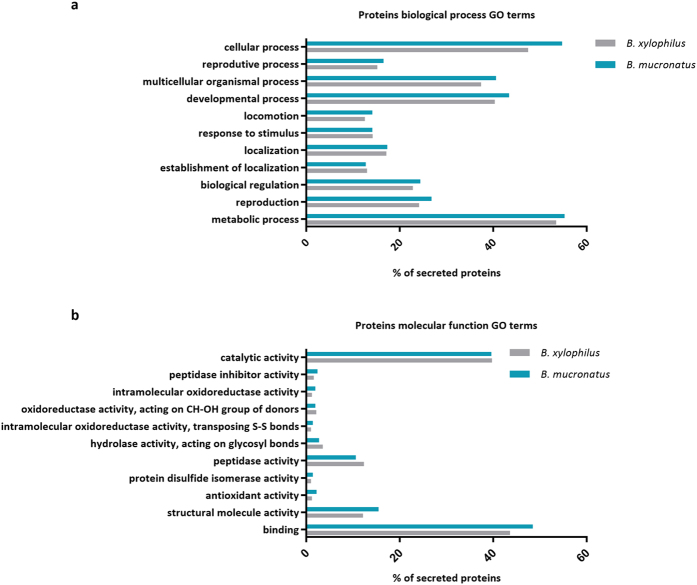
Distribution of *Bursaphelenchus xylophilus* and *B. mucronatus* secreted proteins according to gene ontology (GO) terms. Biological process (**a**) and molecular function (**b**).

Comparative functional analysis of *B. xylophilus* and *B. mucronatus* secretome profiles showed that secreted proteins have a similar GO distribution composition, with a higher percentage of proteins associated with cellular and metabolic processes in biological process GO category (Fig. 3a) and binding and catalytic activity in molecular function GO category (Fig. 3b). At higher levels of molecular function GO terms, only small differences were noted between both secretomes in the percentage of proteins putatively related to pathogenicity, such as peptidase activity and hydrolase activity, acting on glycosyl bonds (Fig. 3b).

**Figure 4:**
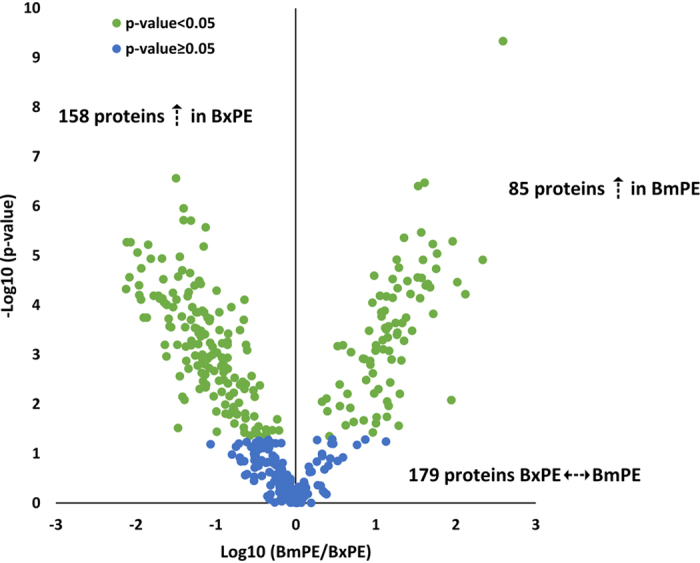
Quantitative proteomic analysis. Volcano plot reflecting the results from the statistical analysis of the 422 proteins quantified among the secretomes of *Bursaphelenchus xylophilus* (BxPE) and *B. mucronatus* (BmPE). Statistical analysis was performed by Student *t*-test and statistical significance was considered for P-values < 0.05.

*Quantitative analysis and identification of differentially secreted proteins.* From the sequential windowed acquisition of all theoretical mass spectra (SWATH-MS) analysis, 446 proteins were quantified, considering proteins with at least one confidence peptide (with a FDR < 0.01%) in at least three out of the six biological replicates per conditions (Supplementary Table S4). According to the normal distribution of the logarithmized quantitative data, statistical analysis was performed by multiple Student *t*-tests for each pair of conditions and proteins with P-values ≥ 0.05 in all the three comparisons were excluded. These correspond to proteins that were at the same levels in the negative control (PE) and in the other two conditions (BxPE and BmPE). According to this evaluation, a total of 422 proteins were quantified and compared between *B. xylophilus* and *B. mucronatus* secretomes (Supplementary Table S5) and from these, 243 proteins were found differentially regulated: 158 and 85 proteins were increased in *B. xylophilus* and *B. mucronatus* secretomes, respectively (Fig. 4).

To gain further insights into the biological differences between *B. xylophilus* and *B. mucronatus*, functional features of the differentially regulated proteins were characterized using GO enrichment analysis, against the entire set of quantitative data. *Bursaphelenchus xylophilus* secretome revealed a strong enrichment in proteins with peptidase activity and also on glycoside hydrolases activity (Table 1). The increased proteins in *B. xylophilus* secretome associated to peptidase activity belong to five catalytic types of peptidases and the glycoside hydrolases increased in *B. xylophilus* secretome were mainly chitinases. Additionally, an enrichment in proteins with peptidase inhibitor activity was also detected, one with serine and three with cysteine -type endopeptidase inhibitor activity (Table 2).

On the other hand, the increased proteins in *B. mucronatus* secretome were mainly related to oxidative stress responses (Table 3) and from these, the proteins related to oxidoreductase activity were associated with 11 different activities (Table 4).

### Evaluation of differential transcript level of cysteine peptidases

**Figure 5:**
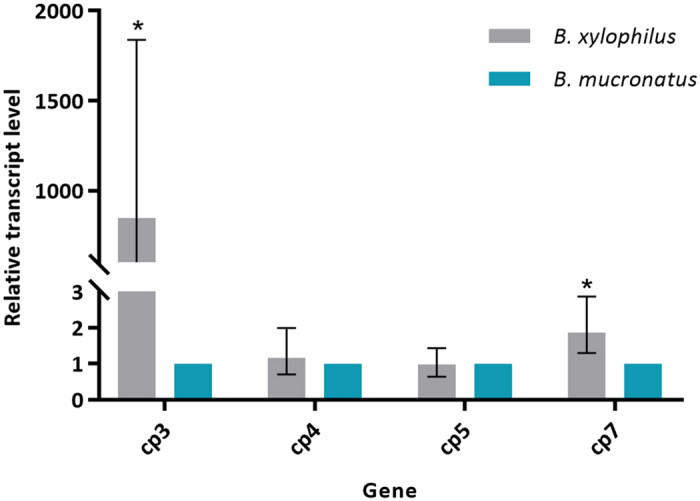
Relative transcript levels of cp3, cp4, cp5 and cp7 genes measured by RT-qPCR. Bars represent the standard error range of three biological replicates and asterisk indicates statistically significant differences (P < 0.036) between *Bursaphelenchus xylophilus* and *B. mucronatus*, determined using the Pair Wise Fixed Reallocation Randomisation Test^©^ in REST software.

Cysteine and serine peptidases constituted the group of proteins with higher representability in the increased proteins in *B. xylophilus* secretome. In order to address whether there was a correlation between transcript level and protein level analyses, RT-qPCR was performed for four cysteine peptidases (CP) selected from the 422 quantified proteins: CP3 and CP7, found increased in *B. xylophilus* secretome, and CP4 and CP5, found unaltered between *B. xylophilus* and *B. mucronatus* secretomes. The RT-qPCR analysis revealed that the cp3 and cp7 transcripts level was significantly (P < 0.036) higher in *B. xylophilus* than in *B. mucronatus*. Moreover, cp4 and cp5 transcript levels were not significantly different (P > 0.05) between both species (Fig. 5). Therefore, the patterns of changes between the two species in transcript levels of these four genes were similar to the changes in protein levels, detected by proteomic analysis.

## Discussion

A database of *B. xylophilus* and *B. mucronatus* transcriptomic data was produced and used for the identification of proteins secreted by these two nematodes under pine tree extract stimulation. A general comparison of transcriptomic profiles did not reveal notorious differences between these species and even when searching for specific groups of proteins, putatively related to nematodes pathogenicity, only small changes were detected. This was mainly in accordance with previous studies using comparative analysis of *B. xylophilus* and *B. mucronatus* transcriptomic data which results indicate that the two species have developed similar molecular mechanisms to adapt to life on pine hosts^13^,^14^. Even though, the use of this combined database in the secretome’s differential analysis allows a higher number of identified protein comparing to those obtained using an annotated *B. xylophilus* protein database derived from genome BioProject PRJEA64437^19^ and thus, constitute an important resource for future genomic and proteomic projects on *Bursaphelenchus* species.

The identified proteins of *B. mucronatus* secretome represent the first proteomic data on the secretome of this species, providing new information about this nematode biology and host interaction. Furthermore, proteomic comparative and quantitative analysis with *B. xylophilus* secretome permitted the identification of proteins detected in different levels in each secretome, reflecting a different response of these nematodes when stimulated by a pine tree extract. No other quantitative proteomic study involving these two species has been presented before. A comparison of secretome profiles of the plant parasitic nematodes *B. xylophilus* and *Meloidogyne incognita* has been previously described and the analysis of GO terms distribution indicated an expansion of peptidases and peptidase inhibitors in *B. xylophilus* secretome^20^. The similar comparative functional analysis of *B. xylophilus* and *B. mucronatus* secretomes, here presented, also revealed a small expansion in peptidases in *B. xylophilus* secretome, nevertheless it was the quantitative analysis of *B. xylophilus* and *B. mucronatus* secretomes that showed significant differences in protein abundances in both secretomes, pointing out groups of proteins possibly responsible for the main differences between these two species pathogenicity. While proteins related to peptidase and glycoside hydrolase activities were detected in higher levels in *B. xylophilus* secretome, in *B. mucronatus* the increased proteins were mainly related to oxidative stress responses.

Peptidases are hydrolytic enzymes that cleave internal peptide bonds within proteins and peptides. They are known to play important functions in all cellular organisms and, in nematodes, peptidases are essential not only during the development processes such as embryogenesis and cuticle remodeling but also in the most critical moments of parasite-host interactions, such as tissue penetration, digestion of host proteins and protection from the host immune system attack^21^. Peptidases can be classified according to their catalytic type and all major types of peptidases have been detected increased in *B. xylophilus* secretome compared to *B. mucronatus* secretome. In other plant parasitic nematodes few reports on secreted peptidases have been presented^22^, however, in animal parasitic nematodes there are many studies describing the secreted peptidases. Cysteine peptidases in animal parasitic nematodes are thought to be involved in tissue penetration, nutrition and defense from the immune system of the host, as well as in moulting. Aspartic peptidases have been described primarily in functions related to the digestion of nutrients and metallopeptidases in functions related to the invasion of host tissues, moulting and digestion of nutrients. The serine peptidases along with the metallopeptidases are believed to play the largest part in the invasion of host tissues^21^. Identified increased peptidases in *B. xylophilus* secretome may well have a key role in this nematode pathogenicity.

On the other hand, glycoside hydrolases are enzymes involved in carbohydrate metabolic process and are part of the known cell-wall degrading enzymes, an important group of enzymes able to break down the carbohydrates that are the essential components of the plant and fungal cell walls. *Bursaphelenchus xylophilus* are known to migrate in resin canals feeding on xylem parenchyma cells of pine trees but also known to feed on fungi colonizing the trees^2^. In the experimental approach, the nematodes stimulated under pine tree extract were recovered from fungi cultures and it is expected that some fungi may be present in the stimulus solution. While chitin and 1,3-beta-glucans are main components of fungi cell wall^23^, cellulose and the other substrates for the identified increased glycoside hydrolases in *B. xylophilus* secretome are components of plant cell walls. Xylem parenchyma cell walls vary among conifer species and in *Pinus* species are mostly thin-walled and unlignified primary walls comprising cellulose, hemicelluloses, pectins and lesser amounts of structural proteins^24^,^25^. The increased of these cell wall degrading enzymes in *B. xylophilus* secretome compared to *B. mucronatus* may reflect a higher capacity of this species to the feed on both plant cells and fungi colonizing trees. Feeding on xylem parenchyma cells *B. xylophilus* causes cell destruction, leading to the development of PWD. Fungi growing in wood tissues of diseased trees provide extra food sources and nematodes develop huge populations whish cause the tree death within few months through damage and blocking of pine tree vascular system^26^.

Additionally, enrichment in peptidase inhibitors were also found in increased proteins of *B. xylophilus* secretome and previous studies on *B. xylophilus* secretome^20^ indicated that the number of secreted peptidase inhibitors in *B. xylophilus* was significantly greater than in other parasitic nematodes. Peptidases are known to play essential roles against pathogens in plant defence system^27^ and overexpression of peptidase genes in the host tree is considered one of the most intense reactions in the case *B. xylophilus* infection^28^. Therefore, these peptidase inhibitors represent an important contribute to the successful evasion of *B. xylophilus* from its host defence response.

The increased proteins in *B. mucronatus* secretome were mainly related to oxidative stress responses and probably play an essential role in nematodes protection from the reactive oxygen species (ROS) accumulated inside the pine trees as a result of the host defence response. Reactive oxygen species are considered to be the first line of defense in plants, oxidizing DNA, proteins and lipids, which causes damage to cellular organelles and inhibits cell functions in plant parasites^29^,^30^. The increased of oxidative stress response proteins gives *B. mucronatus* the ability to propagate and reproduce even in severe environment. The well adaptive properties of this nematode have been previously shown by studies reporting that *B. mucronatus* is found in declining pine trees^31^,^32^ and tends to be more active and fecund, leading to increase nematode population density inside the host tree, at higher temperatures and drought stress^33^,^34^, which are known to enhanced ROS production in the different cellular compartments of the plant cell^35^,^36^. In the present study, antioxidant proteins were also identified in the secretome of *B. xylophilus*. These proteins have been proved as pivotal tools in protecting *B. xylophilus* from ROS and toxic compounds accumulated inside the pine trees^37^,^38^.

Data here presented indicate that it is quite likely that differences in *B. xylophilus* and *B. mucronatus* pathogenicity to pine trees are mainly related to these peptidases, glycoside hydrolases and peptidase inhibitors increased in *B. xylophilus* secretome. This information besides contributing to the clarification of the pathogenicity mechanisms involved in PWD will be of great usefulness for the development of new control strategies for this important forests disease.

## Methods

### Nematodes

Nematodes from Portuguese *B. xylophilus* (BxPt17AS) and *B. mucronatus* (BmPt2) isolates, maintained in cultures of *Botrytis cinerea* grown on Malt Extract Agar medium at 25 °C, were used. Mixed developmental nematode stages grown during 15 days on fungal cultures were collected with distilled water using a 20 μm sieve and washed three times with sterile water.

### *Bursaphelenchus mucronatus* transcriptome sequencing

Total RNA was extracted from ca. 15,000 nematodes as previously described^39^. A fraction of 2.0 μg was used as starting material for cDNA synthesis using the MINT cDNA synthesis kit (Evrogen), where a strategy based on SMART double stranded cDNA synthesis was applied^40^. cDNA was quantified by fluorescence and sequenced in a half a plate of the 454 GS-FLX Titanium system, according to the standard manufacturer’s instructions (Roche-454 Life Sciences). Sequence reads were deposited in the EMBL-EBI European Nucleotide Archive (ENA) under the accession number PRJEB14884.

Sequence processing assembly and annotation was performed as previously described^40^. Prior to the assembly of sequences, the raw reads were processed in order to remove sequences with less than 100 nucleotides and low-quality regions. The ribosomal, mitochondrial and chloroplast reads were also identified and removed from the data set. The reads were then assembled into contigs using 454 Newbler 2.6 (Roche) with the default parameters (40 bp overlap and 90% identity). The translation frame of contigs was assessed through BLASTx searches against Swissprot (e-value ≤ 1e–6) and the corresponding amino acid sequences translated using an in-house script. The contigs without translation were submitted to FrameDP software^41^ and the remaining contigs were analysed with ESTScan^42^. Transcripts resulting from these two last sequence identification steps were searched using BLASTp against the non-redundant NBCI (National Center for Biotechnology Information) database in order to translate the putative proteins. The deduced aminoacid sequences were annotated using InterProScan^43^ which associate each sequence to InterPro protein families or functional domains and predicted the associated GO terms^44^.

### Preparation of *Bursaphelenchus xylophilus* and *B. mucronatus* secreted proteins

A pine wood extract was prepared from two years old *P. pinaster* seedlings using an adaptation of a previously described method^20^ and used as a stimulant for the production of secreted proteins. Briefly, about 15 g of small wood pieces obtained from the stems were soaked in 75 mL of distilled water for 24 h at 4 °C. The collected supernatant solution was passed through a filter paper and then centrifuged through a Vivaspin 5 kDa cutoff membrane (Sartorius Stedim). The pass-through solution containing proteins and metabolites < 5 kDa was collected and re-filtered through a Minisart 0.2 μm cellulose acetate membrane. The obtained solution was used to stimulate the nematodes protein secretion, simulating, *in vitro*, the natural pine stimulus.

Approximately 1 × 10^6^ nematodes of each species were soaked in 5 mL of pine extract for 16 h at 25 °C. Nematodes were then sedimented by centrifugation and the supernatants containing the secreted proteins of *B. xylophilus* (BxPE) and *B. mucronatus* (BmPE) were collected and concentrated to 100 μL with a Vivaspin 5 kDa cutoff membrane. Five mL of pine extract without nematodes were subject to the same conditions and used as control sample (PE). The sedimented nematodes were washed three times in M9 buffer, concentrated via centrifugation and used for RNA extraction, template for reverse transcription quantitative real time PCR (RT-qPCR). Six biological replicates were performed.

### Sample preparation for proteomic analysis

Secretomes previously concentrated were precipitated with Trichloroacetic acid (TCA) - Acetone^45^. The protein pellets were ressuspended in 40 μL of SDS-Sample buffer without bromophenol blue and glycerol^46^, aided by ultrasonication and denaturation at 95 °C. Two μL of each sample were used for protein quantification using the Direct Detect^®^ infrared spectrometer (Millipore) and 60 μg of sample were used in the SWATH-MS analysis. Additionally, the six biological replicates were combined in three pools of 60 μg per condition (two biological replicates per pool) to be used for protein identification and, the same amount of *mal*E-GFP was added.

After denaturation, samples were alkylated with acrylamide and subjected to gel digestion using the short-GeLC approach^47^. The entire lanes were sliced into three parts and processed in separate. Gel pieces were destained, dehydrated and re-hydrated in 70 μL of trypsin (0.01 μg/μL solution in 10 mM ammonium bicarbonate) for 15 min, on ice. After this period, 40 μL of 10 mM ammonium bicarbonate were added and in-gel digestion was performed overnight at room temperature. After the digestion, the formed peptides were extracted from the gel pieces and the peptides extracted from the three fractions of each biological replicate were combined into a single sample for quantitative analysis. All the peptides were dried subjected to SPE using OMIX tips with C18 stationary phase (Agilent Technologies) as recommended by the manufacture. Eluates were dried and ressuspended with a solution of 2% ACN and 0.1% FA containing iRT peptides (Biognosys AG) to be used as internal standards^47^.

### Protein quantification by SWATH-MS

Samples were analysed on a Triple TOF^TM^ 5600 System (ABSciex^®^) in two phases: IDA of the pooled samples and, SWATH-MS acquisition of each individual sample. Peptides were resolved by liquid chromatography (nanoLC Ultra 2D, Eksigent^®^) on a MicroLC column ChromXP^TM^ C18CL (300 μm ID × 15 cm length, 3 μm particles, 120 Å pore size, Eksigent^®^) at 5 μL/min with a multistep gradient: 0–2 min linear gradient from 5 to 10%, 2–45 min linear gradient from 10% to 30% and, 45–46 min to 35% of acetonitrile in 0.1% FA. Peptides were eluted into the mass spectrometer using an electrospray ionization source (DuoSpray^TM^ Source, ABSciex^®^) with a 50 μm internal diameter (ID) stainless steel emitter (NewObjective).

Information-dependent acquisition experiments were performed for each pooled sample. The mass spectrometer was set to scanning full spectra (350–1250 m/z) for 25 ms, followed by up to 100 MS/MS scans (100–1500 m/z from a dynamic accumulation time – minimum 30 ms for precursor above the intensity threshold of 1000 – in order to maintain a cycle time of 3.3 s). Candidate ions with a charge state between +2 and +5 and counts above a minimum threshold of 10 counts per second were isolated for fragmentation and one MS/MS spectra was collected before adding those ions to the exclusion list for 25 seconds (mass spectrometer operated by Analyst^®^ TF 1.7, ABSciex^®^). Rolling collision was used with a collision energy spread of 5. Peptide identification and library generation were performed with Protein Pilot software (v5.1, ABSciex^®^) using the following parameters: i) search against an annotated *B. xylophilus* protein database obtained from Wormbase Parasite derived from BioProject PRJEA64437^19^ or a combined *B. xylophilus* and *B. mucronatus* peptide database obtained from transcriptomic data; ii) acrylamide alkylated cysteines as fixed modification; iii) trypsin as digestion type. An independent False Discovery Rate (FDR) analysis using the target-decoy approach provided with Protein Pilot software was used to assess the quality of the identifications and positive identifications were considered when identified proteins and peptides reached a 5% local FDR^48^,^49^. Transcriptomic data is available for *B. xylophilu*s under the Bioproject PRJNA192936 and for *B. mucronatus* under the submitted Bioproject PRJEB14884.

For SWATH-MS based experiments, the mass spectrometer was operated in a looped product ion mode^50^. The SWATH-MS setup was designed specifically for the set of samples to be analysed (Supplementary Table S6), in order to adapt the SWATH windows to their complexity. A set of 60 windows of variable width was constructed covering the precursor mass range of 350–1250 m/z. A 250 ms survey scan (350–1500 m/z) was acquired at the beginning of each cycle and SWATH MS/MS spectra were collected from 100–1500 m/z for 50 ms resulting in a cycle time of 3.25 s. The collision energy for each window was determined according to the calculation for a charge +2 ion centered upon the window with variable collision energy spread (CES) according with the window.

A specific library of precursor masses and fragment ions was created by combining all files from the IDA experiments, and used for subsequent SWATH processing. Libraries were obtained using Protein Pilot^TM^ software (v5.1, ABSciex^®^) with the same parameters as described above.

Data processing was performed using SWATH^TM^ processing plug-in for PeakView^TM^ (v2.0.01, ABSciex^®^). After retention time adjustment using the *mal*E-GFP peptides, up to 15 peptides, with up to five fragments each, were chosen per protein, and quantitation was attempted for all proteins from the library that were identified below 5% local FDR from ProteinPilot^TM^ searches. Peptides’ confidence threshold was determined based on a FDR analysis using the target-decoy approach and those that met the 1% FDR threshold in at least three of the six biological replicates were retained, and the peak areas of the target fragment ions of those peptides were extracted across the experiments using an extracted-ion chromatogram (XIC) window of 4 min with 100 ppm XIC width.

The levels of the proteins were estimated by summing all the filtered transitions from all the filtered peptides for a given protein and normalized to the total intensity within the same experimental condition. Correlation analysis, performed in InfernoRDN (version 1.1.5581.33355), was used to identify and excluded the less correlated replicates from the posterior analyses. Statistical analysis was performed in MarkerView^TM^ (version 1.2.1.1, ABSciex^®^) using multiple Student *t*-test analysis for comparison between experimental groups. For statistical analysis it was used the normalized protein levels subjected to Log_10_ transformation and statistical significance was considered for P-values < 0.05. Data normality was accessed by the Q-Q plots analysis conducted in InfernoRDN (version 1.1.5581.33355).

### Functional annotation

Gene ontology annotations were performed using the Blast2GO 3.3.5 software^51^ based on the BLAST against the non-redundant protein database NCBI and InterPro database, using the default Blast2GO settings in each step. Gene ontology enrichment analysis of proteins increased in *B. xylophilus* (BxPE) and *B. mucronatus* (BmPE) secretomes against the total number of quantified proteins were performed using Blast2GO with the statistical Fisher’s Exact Test associated and a P-value of 0.05 as cutoff. Gene ontology annotation could be assigned to three different categories: molecular function, that describe the molecular activities of gene products, cellular component that describe where gene products are active and biological process, describing the pathways and larger processes made up of the activities of multiple gene products. MEROPS BLAST search^52^ was also done to accurate the annotation of detected peptidases and peptidase inhibitors after enrichment analysis.

### Relative transcript level by RT-qPCR

The relative transcript abundance of four selected cysteine peptidases (CP3, CP4, CP5 and CP7) was assessed by RT-qPCR. Total RNA was extracted from nematodes. One hundred μL of TRIzol^®^ Reagent (Invitrogen, Waltham, MA, USA) were added to sedimented *B. xylophilus* and *B. mucronatus*, vortexed for two min and subjected to three cycles of freeze and thaw in liquid nitrogen and in a 37 ^ο^C water bath. The RNA was then purified using the Direct-zol RNA MiniPrep kit (Zymo Research Corp.), according to the manufacturer’s instructions and performing the DNase digestion in the column. Extracted RNA quality and quantity were estimated using the spectrophotometer Nanodrop (Thermo Scientific) and the Qubit^®^ 2.0 Fluorometer Qubit (Invitrogen) with the Qubit™ RNA assay kit (Invitrogen). For each sample, 1 μg of extracted RNA was used for cDNA synthesis using SMARTer PCR cDNA Synthesis Kit (Clontech Laboratories Inc.) according to the manufacturer’s instructions. Synthetized cDNA was quantified using Nanodrop and used in qPCR with SybrGreen (Applied Biosystems), according to standard protocols, in the 7500 Fast Real-Time PCR System (Applied Biosystems). The amplification kinetics of each transcript was normalized with the amplification kinetics of the actin and 18 S genes, chosen as endogenous controls. All primers used in qPCR were designed using the Primer Express software (Applied Biosystems) based on the *B. xylophilus* and *B. mucronatus* transcripts sequences, Bioprojects accession numbers PRJNA192936 and PRJEB14884, respectively (Supplementary Table S7). qPCRs were done at 95 °C for 20 s, followed by 40 cycles of 95 °C for 3 s and 60 °C for 30 s. Melting curves analyses were performed and validation experiments were first carried out to ensure equivalent amplification efficiency for all transcripts from both species. The RT-qPCRs were conducted for three biological repetitions, with three technical replicates for each qPCR. Amplification efficiencies and Ct values were determined by the 7500 Fast Real-Time PCR Software v2.0.4 (Applied Biosystems) and the mean Ct values used in the REST software^53^ for relative transcript level and statistically significant differences analysis using the Pair Wise Fixed Reallocation Randomisation Test^©^.

## Additional Information

**How to cite this article**: Cardoso, J. M. S. *et al. Bursaphelenchus xylophilus* and *B. mucronatus* secretomes: a comparative proteomic analysis. *Sci. Rep.* **6**, 39007; doi: 10.1038/srep39007 (2016).

**Publisher's note:** Springer Nature remains neutral with regard to jurisdictional claims in published maps and institutional affiliations.

## Supplementary Material

Supplementary Figures

Supplementary Dataset 1

## Figures and Tables

**Table 1:** Gene ontology (GO) enrichment analysis of the 158 proteins increased in *Bursaphelenchus xylophilus* secretome.

GO ID	GO description	GO category*	P-Value
GO:0008233	peptidase activity	F	1.94E-04
GO:0006508	proteolysis	P	3.90E-04
GO:0070011	peptidase activity, acting on L-amino acid peptides	F	6.94E-04
GO:0004180	carboxypeptidase activity	F	9.52E-04
GO:0016787	hydrolase activity	F	1.15E-03
GO:0004553	hydrolase activity, hydrolyzing O-glycosyl compounds	F	1.33E-03
GO:0004185	serine-type carboxypeptidase activity	F	2.62E-03
GO:0016798	hydrolase activity, acting on glycosyl bonds	F	3.80E-03
GO:1901071	glucosamine-containing compound metabolic process	P	4.25E-03
GO:0006040	amino sugar metabolic process	P	4.25E-03
GO:0006022	aminoglycan metabolic process	P	4.25E-03
GO:0006030	chitin metabolic process	P	4.25E-03
GO:0070008	serine-type exopeptidase activity	F	8.44E-03
GO:0008236	serine-type peptidase activity	F	8.81E-03
GO:0017171	serine hydrolase activity	F	8.81E-03
GO:0003824	catalytic activity	F	1.76E-02
GO:0004175	endopeptidase activity	F	2.39E-02
GO:0008238	exopeptidase activity	F	3.56E-02
GO:0004568	chitinase activity	F	3.85E-02
GO:0008061	chitin binding	F	3.85E-02
GO:0046348	amino sugar catabolic process	P	3.85E-02
GO:1901072	glucosamine-containing compound catabolic process	P	3.85E-02
GO:0009620	response to fungus	P	3.85E-02
GO:0006026	aminoglycan catabolic process	P	3.85E-02
GO:0006032	chitin catabolic process	P	3.85E-02
GO:0030414	peptidase inhibitor activity	F	4.41E-02
GO:0061134	peptidase regulator activity	F	4.41E-02
GO:0044420	extracellular matrix component	C	4.71E-02
GO:0070001	aspartic-type peptidase activity	F	4.71E-02
GO:0004190	aspartic-type endopeptidase activity	F	4.71E-02
GO:0004222	metalloendopeptidase activity	F	4.71E-02
GO:0005604	basement membrane	C	4.71E-02
GO:0051248	negative regulation of protein metabolic process	P	4.71E-02
GO:1901136	carbohydrate derivative catabolic process	P	4.71E-02
GO:0045861	negative regulation of proteolysis	P	4.71E-02
GO:0010466	negative regulation of peptidase activity	P	4.71E-02
GO:0032269	negative regulation of cellular protein metabolic process	P	4.71E-02

Enrichment analysis was performed against all the 442 quantified proteins using a statistical Fisher’s Exact Test associated and a P-value of 0.05 as cutoff. *F refers to molecular function; P to biological process; and C to cellular component.

**Table 2:** Summary of increased peptidases and glycoside hydrolases in *Bursaphelenchus xylophilus* secretome compared to *B. mucronatus* secretome, based on molecular function gene ontology terms.

	Description	#Proteins	Protein ID
Peptidase activity	cysteine-type	9	All_gs454_002631; All_gs454_003203; All_gs454_002316; All_gs454_004450; All_gs454_003244; All_gs454_002475; BmPt2_003216; BmPt2_000767; All_gs454_003032
	serine-type	9	All_gs454_001068; All_gs454_005249; All_gs454_005845; All_gs454_000752; All_gs454_005600; All_gs454_007198; All_gs454_001272; All_gs454_001797; All_gs454_001410
	metallo	6	All_gs454_000155; All_gs454_001243; All_gs454_002836; All_gs454_007821; All_gs454_007450; All_gs454_007798
	aspartic-type	5	All_gs454_002706; All_gs454_002182; All_gs454_002228; All_gs454_002143; All_gs454_002300
	threonine-type	1	BmPt2_001890
glycoside hydrolase activity	chitinase	4	All_gs454_002423; All_gs454_006276; BmPt2_004053; All_gs454_001611
	cellulase	1	All_gs454_006369
	alpha-1,4-glucosidase	1	All_gs454_000105
	alpha-galactosidase	1	All_gs454_002135
	fucosidase	1	All_gs454_002563
	glucan endo-1,3-beta-D-glucosidase	1	All_gs454_005432
endopeptidase inhibitor activity	serine-type	1	All_gs454_001641
	cysteine-type	3	All_gs454_009328; All_gs454_014827; All_gs454_008917

**Table 3:** Gene ontology (GO) enrichment analysis of the 85 proteins increased in *Bursaphelenchus mucronatus* secretome.

GO ID	GO description	GO category*	P-Value
GO:0009636	response to toxic substance	P	3.12E-03
GO:0016491	oxidoreductase activity	F	3.76E-03
GO:1901700	response to oxygen-containing compound	P	5.47E-03
GO:0098754	Detoxification	P	1.17E-02
GO:0098869	cellular oxidant detoxification	P	1.17E-02
GO:0016209	antioxidant activity	F	1.17E-02
GO:0000302	response to reactive oxygen species	P	1.17E-02
GO:1990748	cellular detoxification	P	1.17E-02
GO:0006979	response to oxidative stress	P	1.37E-02
GO:0032535	regulation of cellular component size	P	1.39E-02
GO:0090066	regulation of anatomical structure size	P	1.39E-02
GO:0065008	regulation of biological quality	P	1.54E-02
GO:0055114	oxidation-reduction process	P	1.98E-02
GO:0060548	negative regulation of cell death	P	2.52E-02
GO:0044710	single-organism metabolic process	P	2.65E-02
GO:0065007	biological regulation	P	2.78E-02
GO:0032787	monocarboxylic acid metabolic process	P	3.81E-02
GO:0010035	response to inorganic substance	P	3.98E-02
GO:0051128	regulation of cellular component organization	P	3.98E-02
GO:0050793	regulation of dkevelopmental process	P	4.41E-02
GO:0051239	regulation of multicellular organismal process	P	4.41E-02
GO:0050789	regulation of biological process	P	4.58E-02

Enrichment analysis was performed against all the 442 quantified proteins using a statistical Fisher’s Exact Test associated and a P-value of 0.05 as cutoff. *F refers to molecular function; P to biological process; and C to cellular component.

**Table 4:** Summary of increased oxidoreductases in *Bursaphelenchus mucronatus* secretome compared to *B. xylophilus* secretome, based on molecular function gene ontology terms.

	Description	#Proteins	Protein ID
Oxireductase activity	superoxide dismutase	2	BmPt2_003588; BmPt2_0004784
	ferroxidase	1	BmPt2_003434
	peroxiredoxine	1	BmPt2_002820
	glutathione peroxidase	1	BmPt2_002173
	thioredoxin	1	BmPt2_001460
	aldo keto reductase	1	BmPt2_001300
	4-hydroxyphenylpyruvate dioxygenase	1	BmPt2_000992
	glyceraldeyde-3-phosphate dehydrogenase	1	BmPt2_000845
	alcohol dehydrogenase	1	BmPt2_000771
	glutathione-dissulfide reductase	1	BmPt2_000185
	dissulfide-isomerase domain	1	BmPt2_000117
