# Supplementary material for: Bursaphelenchus xylophilus and B. mucronatus secretomes: a comparative proteomic analysis
Source: Sci Rep. 2016 Dec 12;6:39007. doi: 10.1038/srep39007 (PMC5150578; doi:10.1038/srep39007)
Supplement: Supplementary Figures [file srep39007-s1.pdf]

***Bursaphelenchus xylophilus* and *B. mucronatus* secretomes: a comparative proteomic analysis**

Joana M.S. Cardoso, Sandra I. Anjo, Luís Fonseca, Conceição Egas, Bruno Manadas, Isabel Abrantes

Supplementary Figure S1

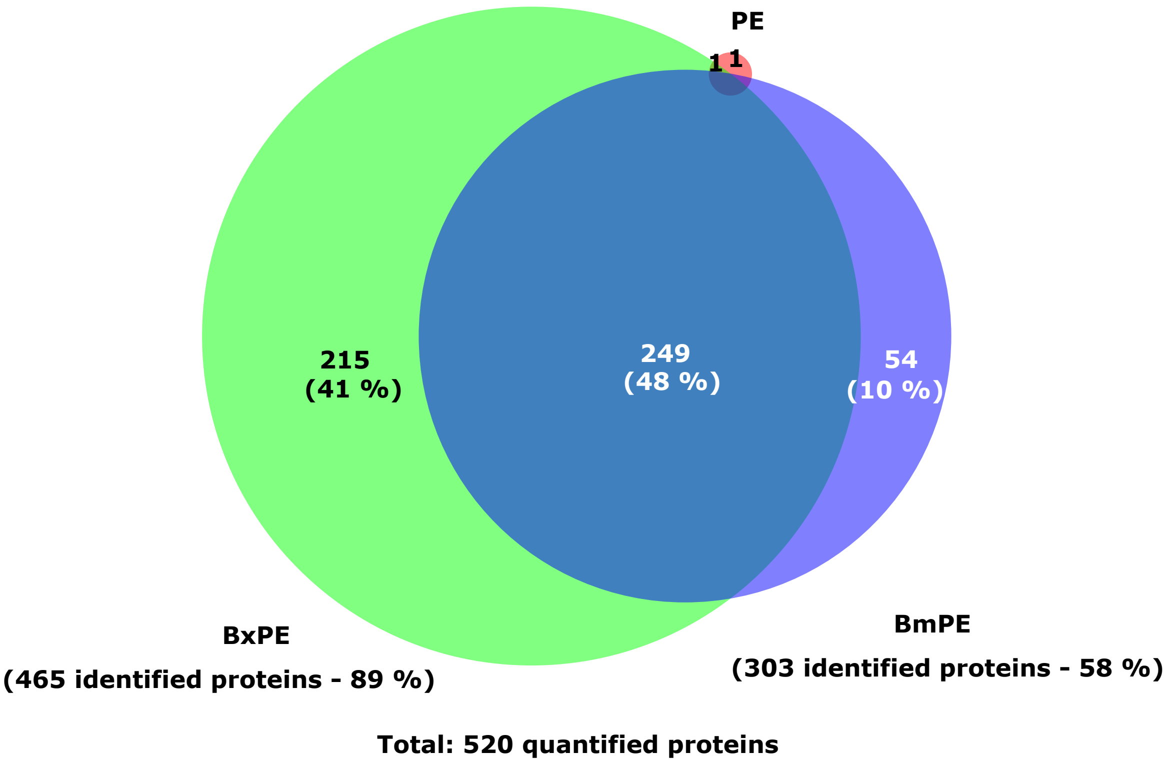

Figure S1.Venn diagram showing the distribution of identified proteins after information-dependent acquisition (IDA) experiments using the genomic derived database. *Bursaphelenchus xylophilus* secretome (BxPE), *B. mucronatus* secretome (BmPE) and pine extract (PE). Protein identifications were obtained by combining the results of the three pooled samples of each condition.

Supplementary Figure S2

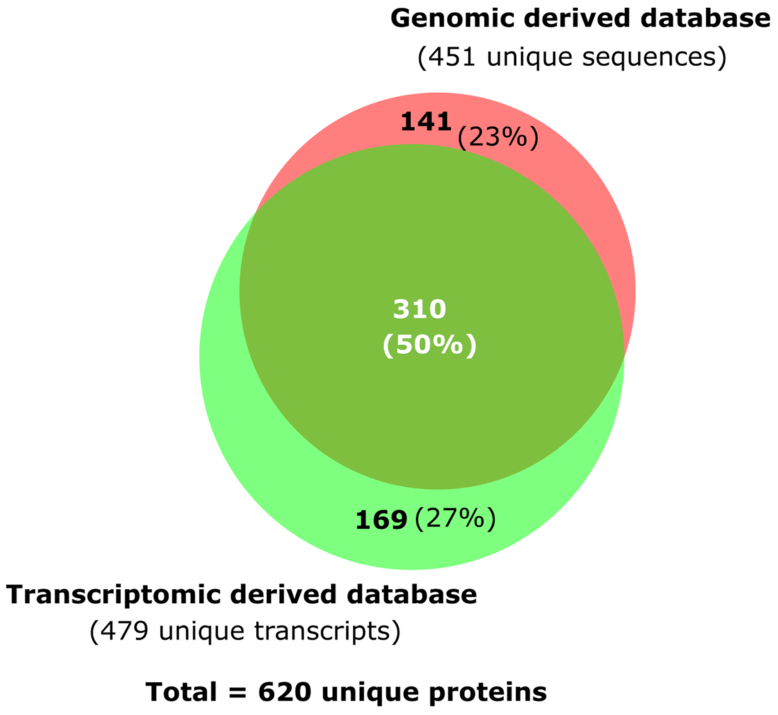

Figure S2. Venn diagram comparing the proteins identified in the genomic derived database and in the transcriptomic derived database. In order to accurately compare the overall proteins identified using the non-curated databases used in the present work, a single search was performed by combining the results of all the samples acquired against a single database composed by the combination of the genomic and the *B. xylophilus* and *B. mucronatus* transcriptomic derived databases. In this sense, sequence matching between the genomic and transcriptomic derived databases were performed based on the peptides identified, and the common sequences were reflected in the proteins belonging to the same protein group (i.e., the proteins justified by the same set of identified peptides). See details in Supplementary Table S3.
